# Evaluation of iTRAQ and SWATH-MS for the Quantification of Proteins Associated with Insulin Resistance in Human Duodenal Biopsy Samples

**DOI:** 10.1371/journal.pone.0125934

**Published:** 2015-05-07

**Authors:** Sylvie Bourassa, Frédéric Fournier, Benjamin Nehmé, Isabelle Kelly, André Tremblay, Valéry Lemelin, Benoit Lamarche, Patrick Couture, Arnaud Droit

**Affiliations:** 1 Proteomics Center, CHU de Québec Research Center and Department of Molecular Medicine, Laval University, Quebec, Canada; 2 Lipid Research Center, Centre Hospitalier de l’Université Laval Research Center, Laval University, Quebec, Canada; USDA-ARS, UNITED STATES

## Abstract

Insulin resistance (IR) is associated with increased production of triglyceride-rich lipoproteins of intestinal origin. In order to assess whether insulin resistance affects the proteins involved in lipid metabolism, we used two mass spectrometry based quantitative proteomics techniques to compare the intestinal proteome of 14 IR patients to that of 15 insulin sensitive (IS) control patients matched for age and waist circumference. A total of 3886 proteins were identified by the iTRAQ (Isobaric Tags for Relative and Absolute Quantitation) mass spectrometry approach and 2290 by the SWATH-MS strategy (Serial Window Acquisition of Theoretical Spectra). Using these two methods, 208 common proteins were identified with a confidence corresponding to FDR < 1%, and quantified with p-value < 0.05. The quantification of those 208 proteins has a Pearson correlation coefficient (r^2^) of 0.728 across the two techniques. Gene Ontology analyses of the differentially expressed proteins revealed that annotations related to lipid metabolic process and oxidation reduction process are overly represented in the set of under-expressed proteins in IR subjects. Furthermore, both methods quantified proteins of relevance to IR. These data also showed that SWATH-MS is a promising and compelling alternative to iTRAQ for protein quantitation of complex mixtures.

## Introduction

Insulin resistance (IR) is associated with dyslipidemia, which contributes to the pathogenesis of atherosclerosis and increases the risk of cardiovascular disease [1]. Studies have also consistently supported the concept that inducing IR in various animal models is associated with significant up-regulation of the expression of key intestinal genes involved in lipid and lipoprotein metabolism [2, 3]. We recently showed that IR in humans was associated with a significant down-regulation of several key genes involved in intestinal fatty acid and lipoprotein metabolism [4]. However, no study has yet examined duodenal protein expression using global quantitative proteomics analyses from IR vs insulin sensitive (IS) patients.

A number of techniques and strategies can be used for the relative comparison of protein expression between different conditions. Isotopic labeling and label-free methods are currently used for this task. There are some publications and reviews comparing both methods in the last years [5–10], but no consensus has been made on which method is superior, each of them having its own strengths and weaknesses.

The most popular labeling approaches are iTRAQ (isobaric tag for relative and absolute quantitation), SILAC (stable isotope labeling by amino acids in cell culture) and TMT (tandem mass tag). The iTRAQ reagents as well as the TMT reagents react with peptide amino-termini or lysine residues, and hence label most peptides and proteins of the cells. Upon collision-induced dissociation (CID) or higher-energy collisional dissociation (HCD), iTRAQ or TMT reporter ions (4 or 8 for iTRAQ and 6 or 10 for TMT) are released in the MS/MS spectra. The intensity of these peaks will be used for the relative quantification of peptides and proteins.

In recent years, label-free quantification based on precursor signal intensity has gained popularity because of its fast and low-cost measurement. Peak intensity based comparative LC/MS and spectral count based LC-MS/MS are the most commonly used label-free quantification methods [11–13]. New label-free mass spectrometry strategies have recently emerged and hold great promise. An interesting review [6] present the advantages and limitations of the data-independent analysis (DIA) and hyper reaction monitoring. Selected Reaction Monitoring (SRM) and Multiple Reaction Monitoring enable the quantification of predetermined proteins by targeting specific peptides (detected in previous experiments). The targeted approach is very specific, reproducible, sensitive, and allows either relative or absolute quantification. However, this technique is also time consuming since optimization work must be done prior analysis to obtain optimal specificity and sensitivity.

Most label-based and label-free approaches use data-dependent acquisition (DDA) where a survey scan is used to determine which precursor will be selected for product ion scanning [6, 14]. However, with DDA, low intensity ions are often missed and thus cannot be quantified [14]. The SWATH approach [15] (Sequential Window Acquisition of all Theoretical Mass Spectra) circumvent this effect since it uses DIA for the quantification. DIA operates without any prior knowledge of the precursor ion to trigger acquisition of fragment ion spectra. Indeed, data are acquired by repeatedly cycling through predefined sequential windows over the whole chromatographic elution range generating a complete recording of all analytes in the sample. Despite its present incompatibility with conventional database searching, SWATH is a nice tool for quantification of a large number of proteins from a complex mixture. Unlike MRM, SWATH has the capability and flexibility to re-mine the data post-analytically.

There are some studies comparing labeled and label-free strategies. iTRAQ has been compared to gel and label-free LC-MS/MS [8] on the *Methylocella silvestris* bacterium. The study concludes that the three methods were comparable for the number of proteins identified if single peptides were used for protein identification. There is also a good correlation between the relative quantification by iTRAQ and label-free LC-MS/MS. However, the experiments were not conducted on the same instrument (QTOF global Ultima compared with QTOF premier).

Another group compared iTRAQ and peak-intensity-based label-free approaches on *Chlamydomonas reinhardtii* strains on the LTQ orbitrap velos platform [10]. Quantification was calculated by summing the abundances of all peptides of the respective proteins. A scatter plot comparing the quantified proteins by the two methods showed a good correlation coefficient of 0.78. However, label-free approach led to more protein being identified and quantified, but the reproducibility was better with iTRAQ.

A recent article [9] comparing iTRAQ and label-free proteomics in human lung adenovirus infection suggested that the label-free method is more accurate than the iTRAQ method. However, the comparison was performed on different instruments (MALDI-TOF-TOF for iTRAQ and LTQ-FT-ICR mass spectrometer for label-free). A higher dynamic range was found with the label-free approach.

Lambert et al [16] showed some comparisons of SWATH data and iTRAQ. They used iTRAQ and AP-Western for validation of the SWATH results. More recently, Zhang et al [17] used iTRAQ and SWATH for the identification of cancer-related proteins in metastatic non-small-cell lung cancer (NSCLC). Using both methods, they found that CD109 could be a potential biomarker for NSCLC.

In this study, we evaluated and compared the iTRAQ labeling technique and the label-free SWATH-MS strategy in duodenal biopsy of IR vs IS subjects. This picture of the differential proteome corroborates previous positions about the effects of insulin resistance and raises interesting hypothesis about the implication of other proteins such as PACAP and MARCKS in the onset of insulin resistance in duodenum.

## Materials and Methods

### Patient samples and duodenal biopsy

Fourteen nondiabetic, insulin-resistant (IR) males and fifteen insulin-sensitive (IS) males matched for age and waist circumference were recruited in the Quebec City area to participate in the study. IR subjects had to have plasma triglyceride (TG) levels > 1.7 mmol/L, HDL-C < 1.1 mmol/L, plasma insulin levels > 90 ρmol/L and a waist circumference > 94 cm. Subjects were excluded if they had elevated blood pressure, monogenic hyperlipidemia such as familial hypercholesterolemia, plasma TG levels > 4.5 mmol/L, a recent history of alcohol or drug abuse, diabetes mellitus or a history of cancer. Furthermore, all participants were unrelated at the first and second degrees. The research protocol was approved by the Laval University Medical Center ethical review committee and written informed consent was obtained from each subject. Biopsies were obtained from the second portion of the duodenum during gastro-duodenoscopy. Six biopsy samples were collected using multiple sample single-use biopsy forceps and immediately flash frozen in liquid nitrogen and stored at -80°C before protein extraction.

### Protein extraction

Frozen duodenal tissue biopsies (15 IS and 14 IR patients) were weighted (6 to 20mg), and disrupted using a mortar and pestle. Samples were kept frozen on dry ice, and grinded to fine powder. Then lysis buffer (50mM ammonium bicarbonate, 50mM dithiothreitol (DTT), 0.5% sodium deoxycholate (SDC)) containing protease inhibitors cocktail (Roche) was added, and the sample preparation was homogenized on ice by sonication with a Sonic Dismembrator (Fisher) with 1 second pulse (20 times). Samples were centrifuged 10 min at 16000g. The supernatants were mixed with 5 volumes of acetone (stored at -20°C) and incubated overnight at -20°C. Precipitated proteins were centrifuged 15min at 16000g. Protein pellets were air dried, and then resuspended in 0.5M Triethylammonium bicarbonate (TEAB) containing 0.5% SDC. Finally, the protein concentration of each sample was determined by colorimetric Bradford assay.

### iTRAQ sample labelling and isoelectric focusing (IEF)

**Fig 1:**
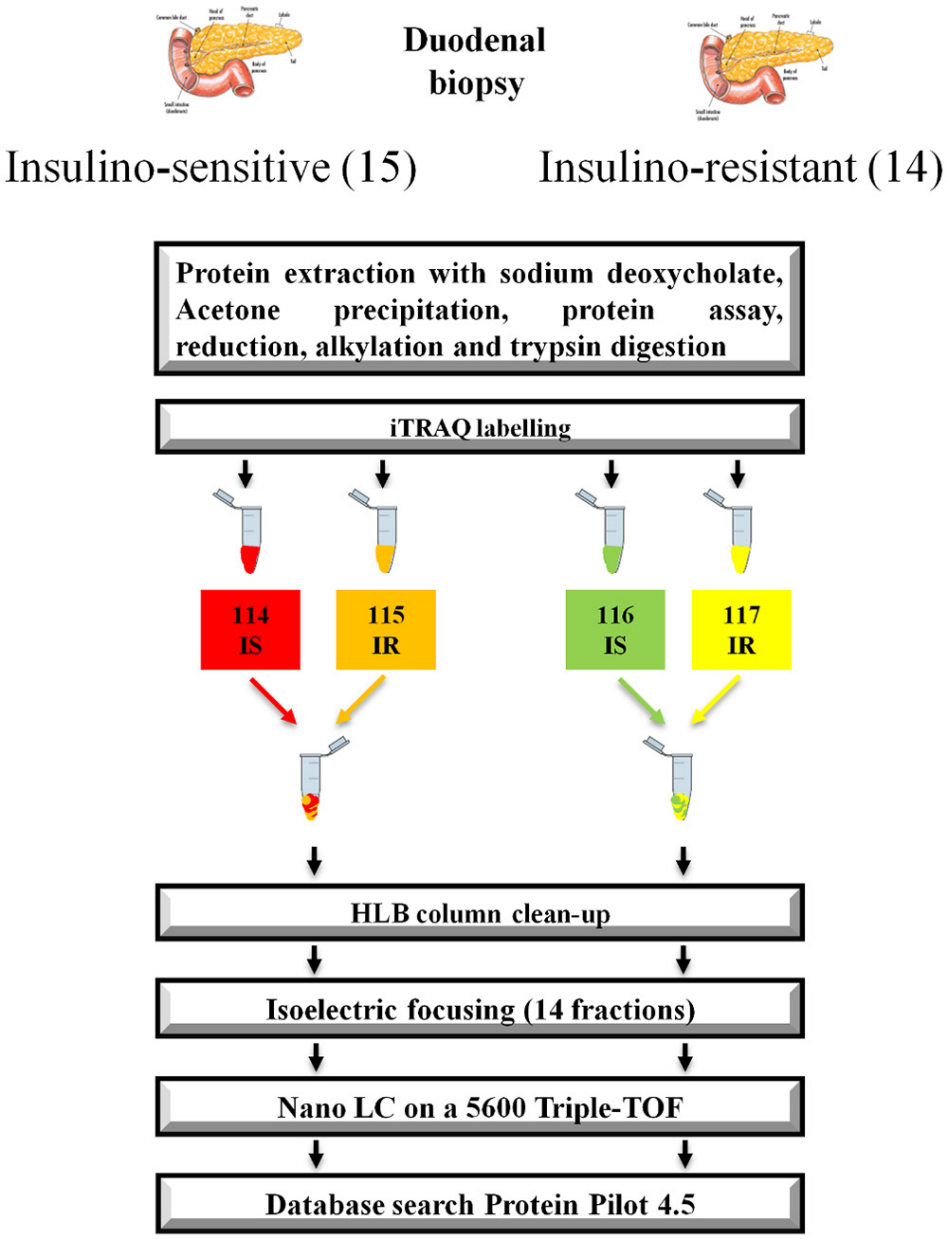
Workflow for discovery (iTRAQ) experiment. Duodenum biopsy samples were obtained from 15 IS volunteers and 14 IR patients. Samples were pooled and two technical replicates were generated.

Equal amounts of protein (50μg) from each control (IS) or IR sample were combined to give a control group and a IR group, respectively (Fig 1). Experimentation was performed in duplicates. Protein concentrations in pooled control (IS) and IR groups were determined using the Bradford assay. Then a total of 100 μg of protein per group was used for iTRAQ labeling. TEAB and SDC were added to each sample to reach a final concentration of 0.5 M and 0.5%, respectively. Proteins were then reduced and alkylated according to the iTRAQ kit manufacturer’s instructions (AB SCIEX). Samples were digested with trypsin (Sequence grade Modified, Promega) using 1:30 ratio overnight at 37°C. After digestion, peptides were acidified to precipitate SDC, and then purified with an oasis HLB cartridge (1cc, 10mg, Water Corp.) and lyophilized. Dried peptides were dissolved in 30μl 0.5M TEAB and labeled with iTRAQ label reagent (AB SCIEX). 4-plex labeling was performed for 2 h at room temperature in the dark. Labeled peptides were combined in one tube and dried with the SpeedVac. Samples were cleaned up using HLB cartridge (Water Corp.). Samples were dried and reconstituted in 200μl HPLC water and 1/100 ampholytes pH 3–10 (Biorad). Then peptides were fractionated with 7cm IPG strips pH 3–10 using IEF. IPG (immobilized pH gradient) strips were passively rehydrated 5 hours. Focusing was performed according to the following protocol: 0–250 V (gradient over 15 min); 250–4000 V (gradient over 2 h); 4000 V (fixed, until a total of 10000 Vh). Strips were cut in 14 fractions and peptides were extracted in 2% ACN—1%FA solution followed by 50% ACN—1% FA. Finally, fractions were dried with the SpeedVac.

### LC-MS/MS acquisition for iTRAQ samples

Fractions collected from the isoelectric focusing were resuspended in 25 ul 0.1% formic acid. Mass spectrometry analysis was performed on a TripleTOF 5600 mass spectrometer fitted with a nanospray III ion source (AB SCIEX, Concord, ON) and coupled to an Agilent 1200 HPLC (Agilent, California). Two ul samples were injected by the Agilent 1200 autosampler onto a trapping column (Zorbax 300SB-C18) 5 u, 5 x 0.3mm at ten ul/min for desalting then onto a 0.075 mm (internal diameter) self-packed PicoFrit column (New Objective) packed with a isopropanol slurry of 5um Jupiter C18 (Phenomenex) stationary phase using a pressure vessel (Proxeon) set at 700 psi. The length of the column was 15 cm. Samples were run using a 90 min gradient from 5–35% solvent B (solvent A 0.1% formic acid in water; solvent B: 0.1% formic acid in acetonitrile) at a flow rate of 300 nl/min. Data were acquired using an ion spray voltage of 2.4 kV, curtain gaz of 30 psi, nebulizer gaz of 8 psi and an interface heater temperature of 125°C. A DDA method was set up with the MS survey range set between 400 amu and 1250 amu (250msec) followed by dependent MS/MS scans with a mass range set between 100 and 1800 amu (50m sec) of the 20 most intense ions in the high sensitivity mode with a 2+ to 5+ charge state. Dynamic exclusion was set for a period of 12 sec and a tolerance of 100 ppm. Rolling collision energy was used and iTRAQ reagent collision energy adjustment was on.

Data files were submitted for simultaneous searches using Protein Pilot version 4.5 software (AB SCIEX) utilizing the Paragon and Progroup algortihms [18] and the integrated false discovery rate (FDR) analysis function [19]. Protein Pilot was set up to search the uniprot ‘complete proteome’ human proteins database (84848 sequences) with MMTS as a fixed modification on cysteine. Variable peptide modifications included methionine (M) oxidation and iTRAQ labeling of the N-terminal, lysine (K) and tyrosine (Y). Automatic normalization of quantitative data (bias correction) was performed to correct any experimental or systematic bias.

The detected protein threshold in the software was set to the value which corresponded to 1% FDR. The following criteria were required to consider a protein for further statistical analysis: the *p*-value of the protein quantitation had to be ≤ 0.05. All data files are available in the PRIDE database (submission number: 1-20141114-204).

### Library generation for SWATH analysis

200 ug of tryptic peptides from IS and IR samples in which we spiked 20 pmol of bovine serum albumin peptides (as an internal standard) were fractionated by IEF into 14 fractions (as described above). Two hundred fifty ng from each fraction was injected on the TripleTOF 5600 (AB SCIEX) in DDA mode using the same chromatography conditions and the same acquisition parameters as used for iTRAQ (except for the extra CE for iTRAQ) and then searched against the uniprot ‘Complete Proteome’ human database (release of March 2013, 84848 sequences) with Protein Pilot 4.5. The resulting protein pilot. group file was used to generate the library which was used for SWATH processing and quantification.

### SWATH analysis

**Fig 2:**
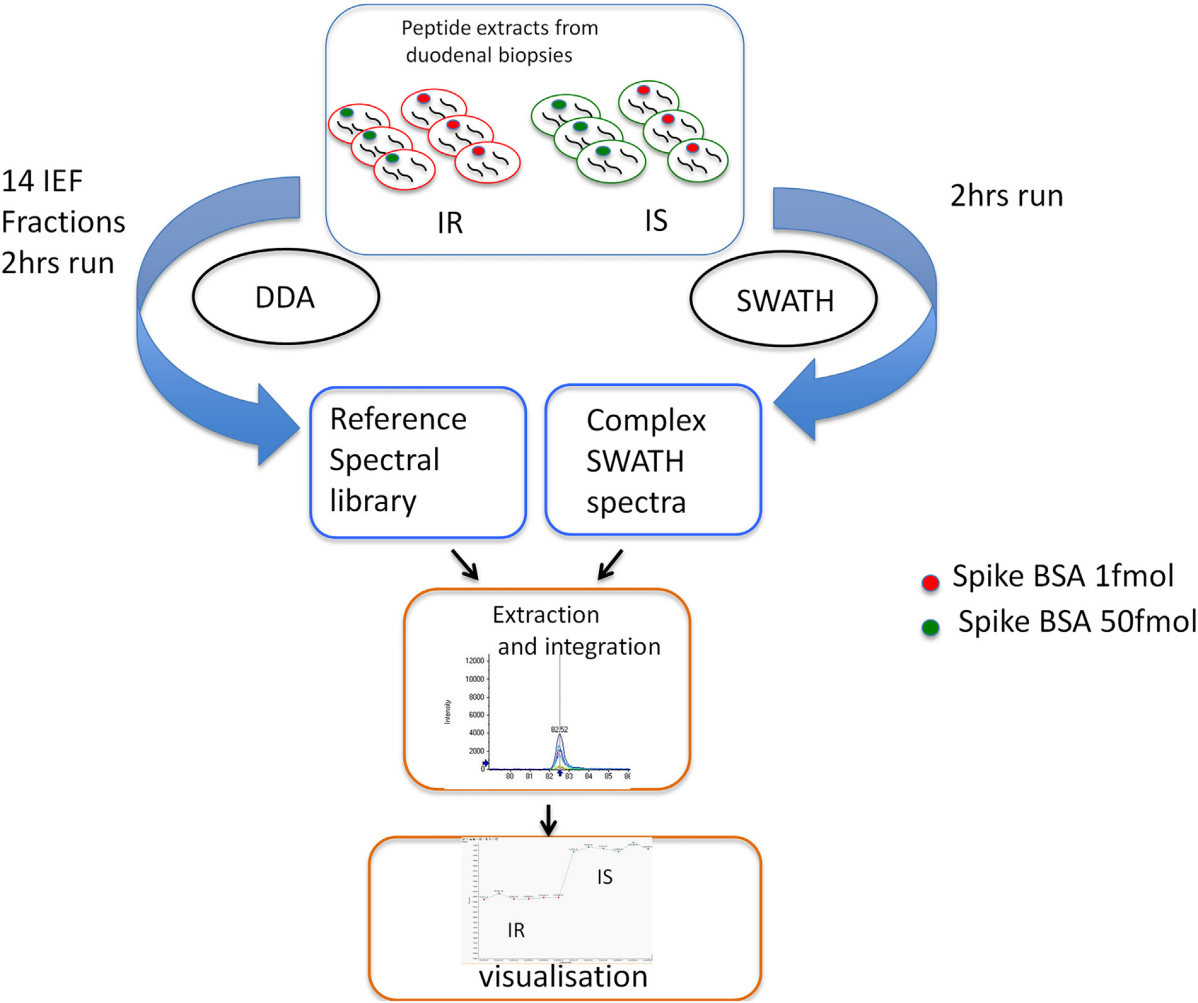
SWATH workflow. Each condition is injected separately in a DDA mode to build the spectral library and in a SWATH mode. The spectral library built from the DDA runs is then used by PeakView and MarkerView to extract the peptide and the quantification information in each of the SWATH runs. BSA 1 fmol and 50 fmol was spiked in each SWATH run as an internal standard.

Tryptic peptide samples from IR (200ng) and IS (200ng) were injected in 6 replicates in data independent acquisition (DIA) mode for SWATH analysis. 3 replicates of IR and IS contained 10 fmol of BSA and the 3 other replicates of IR and IS contained 50fmol of BSA (Fig 2). The SWATH conditions were essentially the same as in Gillet et al. [15] with the same chromatographic conditions described in the iTRAQ experiment. The mass spectrometer was operated with a 50 ms TOF MS scan followed by product ion mode of 100 ms 24 x 25 amu isolation window covering a mass range of 400–1000 with cycle time of 2.5 sec. An overlap of 1 Da between each SWATH was used. Material eluted in a linear gradient of 5–35% acetonitrile over 90 min as in the generation of the SWATH library.

### SWATH Data Processing

Data were processed with PeakView 2.0 and MarkerView 1.2. The result file from the DDA experiment used for the library generation was imported into PeakView with a protein FDR threshold of 1%. Ten peptides per protein and 10 transitions per peptide were extracted from the SWATH files. Shared peptides were excluded as well as peptides with modifications. Peptides with FDR lower than 1.0% were exported in MarkerView for the t-test.

### MRM analysis

In order to select tryptic peptides that are the most suitable for sensitive and selective protein detection, MRM analysis were performed on tryptic peptides unique to the studied proteins with length ranging from 5 to 25 amino acids. Peptides containing methionine and cysteine were eliminated. Peptides were selected using Skyline v2.5 [20] based on peak shape and intensity.

Purified synthetic peptides containing ^13^C_6_ Lys and ^13^C_6_ Arg were obtained from JPT (Germany) and reconstituted in 0.1% formic acid to a final concentration of 500 pmol/μL. A solution containing 10 fmol/μL of each peptide was prepared from the stock solutions and used to reconstitute the samples after tryptic digestion for relative quantification. 1.25 ug of peptides (in 5 ul) were analyzed on a AB SCIEX 5500QTRAP hybrid triple quadrupole/linear ion trap mass spectrometer equipped with an Eksigent nanoLC AS2 cHiPLC nanoflex controlled by Analyst 1.6 and with a nanospray ionization source. MS analysis was conducted in positive ion mode with an ion spray voltage of 2300V. Peptides were desalted on a 200um x 0.5 mm chip trap column packed with ChromXP C18, 3 um, (Eksigent) at 2 ul/min of Solvent A (formic 0.1%) then switched in line at a flow rate of 300 nL/min on a 75um x 15 cm chip column packed with ChromXP C18, 3 um (Eksigent) with a 20 min linear gradient from 5 to 25% of solvent B (ACN 0.1% FA), then a 2 min linear gradient from 25 to 80% B, followed by a 8 min linear gradient. Nebulizer gas was set to 8 (Gas1), curtain gas to 20, heater to 150°C and declustering potential (DP) to 70 V. LC-MRM/MS analyses were performed using three transitions on two peptides for each of the target proteins and quantification done with MultiQuant 2.1 was based on the relative areas of the SIS and endogenous peptides. The MRM transition that gave the highest area counts was used for the quantitation, with the other two transitions acting as qualifier transitions to confirm peptide retention times and the fragment ion ratios. A blank solvent injection was run between biological samples to prevent sample carryover on the HPLC column and the samples were injected in random order. Samples were analyzed in duplicate. Samples containing 5 fmol of digested BSA were injected periodically in order to confirm system stability.

### Bioinformatics

Statistical analysis was done on the R/Bioconductor plateform [21] using R v3.0.1 and Bioconductor release 2.12. For the gene ontology analyses, the software packages GOstats v2.28 [22] and biomaRt v2.18 [23] were used as well as the annotation packages GO.db and org.Hs.eg.db (both at v2.10.1). The enrichment in annotations was calculated for the list of under-expressed and over-expressed proteins for every GO annotation in the three ontologies (cellular component, molecular function and biological process). The selection of the key GO terms was done firstly on the basis of p-value of the annotation enrichment (min p-value of 1e-2), secondly on the basis of number of differential proteins described by the GO term (minimum 2), and thirdly on the basis of the relevance of the annotation to the current study. Whereas the two first steps are fully automated, the third step implies a manual choice guided by the context of the current experiment.

## Results and Discussion

In this study, we evaluated and compared two quantitative proteomics strategies, the iTRAQ labeling technique and the label-free SWATH-MS strategy, to discover the proteins that are potentially associated with insulin resistance in duodenal biopsy samples from IR and IS subjects.

### Protein identification and quantification

Tryptic peptides of proteins extracted from duodenal biopsy samples of IS and IR patients were labeled with the four-plex iTRAQ reagents in duplicates. After labeling, sample IR #1 was pooled with sample IS#1 and sample IR#2 was pooled with sample IS#2 creating two replicates. Those two technical replicates were treated separately for the subsequent steps according to Fig 1.

Using Protein Pilot, a total of 3886 protein groups were identified in at least one iTRAQ replicates with global FDR < 1% (S1 Table). Three hundred of these proteins were quantified with a p-value <0.05 (S2 Table and S3 Table) as calculated by Protein Pilot based on two-tailed t-tests where the degree of freedom is equal to the number of distinct peptide minus one.

For the SWATH analysis, a spectral library of 2290 proteins was created with FDR <1% by injecting a new set of 14 fractions with a Data Dependent Acquistion method on the 5600 triple-TOF mass spectrometer (Fig 2 and S4 Table).

A SWATH experiment was performed on 6 replicates of IS and 6 of IR from which 3 samples of IR and 3 of IS were spiked with BSA 1fmol and the 3 others with BSA 50 fmol (Fig 2).

The library and the 12 SWATH files were uploaded into PeakView. One thousand three hundred twenty-six proteins with at least one peptide with a FDR < 1% were exported into MarkerView. Marker View performed t-test comparing IS and IR groups for each protein: 798 proteins were quantified with p-value below 0.05 (S5 Table).

### Comparison of the differentially expressed proteins between iTRAQ and SWATH

In this study, more proteins were detected by iTRAQ than in the SWATH experiment since the iTRAQ protein identifications came from the two replicates of 14 fractions whereas the SWATH library was created using 14 fractions. In order to increase the SWATH library, the iTRAQ dataset was used to build the SWATH library by using a conversion option designed for this in the PeakView software. Unfortunately, we observed that the peptides detected from the iTRAQ experiment often produced ions of higher charge state than the same peptide from a non-labeled experiment and were not the ones giving the best signals in the SWATH experiment. This probably explains why using the data from the iTRAQ experiment as a library for SWATH produced sub-optimal results. To increase the size of the SWATH library, more injections from duodenal extract would be needed.

**Fig 3:**
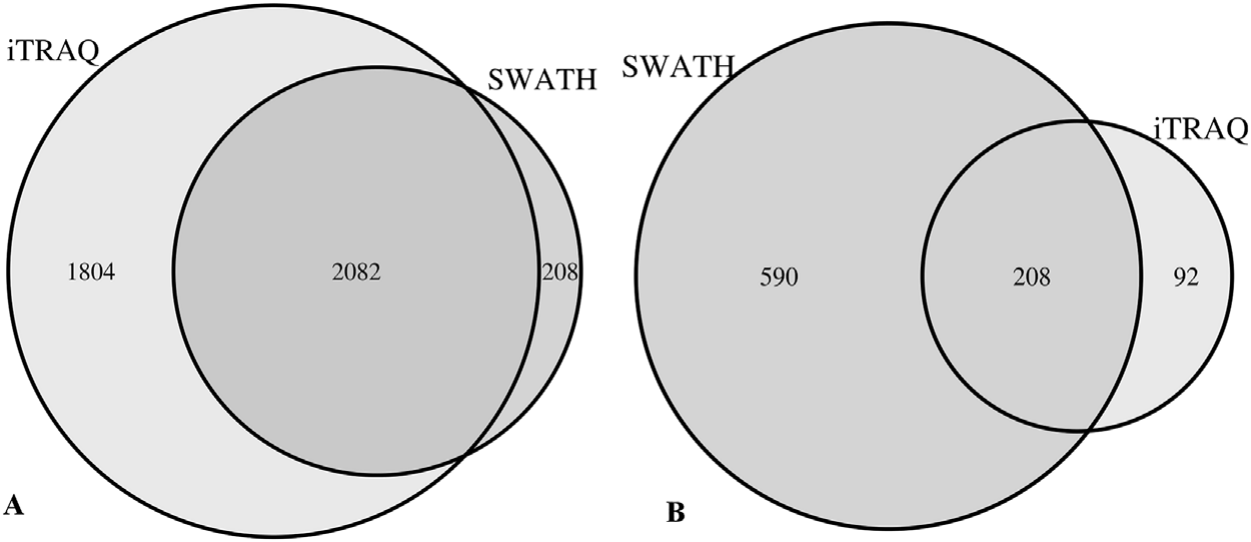
Venn diagram showing the comparison between iTRAQ and SWATH. (A) Comparison in the number of protein identification at FDR<1% (B) Comparison in the number of proteins quantified at p-value <0.05.

Two thousand eighty-two proteins were commonly identified between the two techniques at FDR <1% (Fig 3A) Of these, 208 proteins have a log ratio different from 0 and a p-value <0.05 (as calculated by Protein Pilot for iTRAQ and Marker View for SWATH) (Fig 3B and S6 Table).

### Correlation Analysis for iTRAQ-Based versus SWATH-MS quantitation

**Fig 4:**
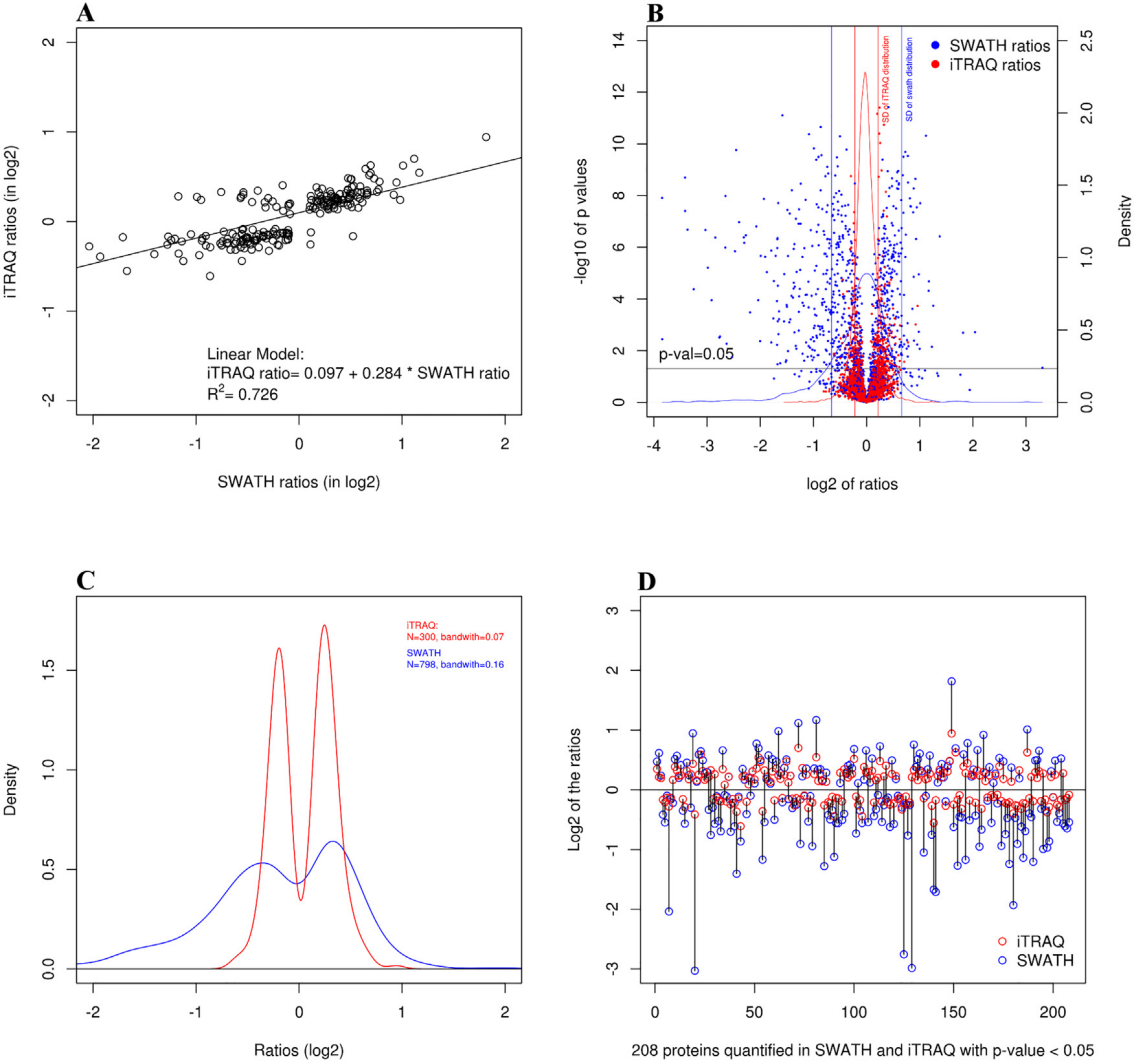
Comparison of iTRAQ and SWATH using different approaches A) Scatter plot analysis of the 208 quantified proteins by iTRAQ and SWATH B) Volcano plot of SWATH ratios vs iTRAQ C) Comparison between SWATH and iTRAQ ratios for the 208 quantified proteins D) Density plot of iTRAQ and SWATH ratios for all differential ratios with p-value <0.05.

We next assessed the level of correlation between quantification by iTRAQ-label and by SWATH-MS (Fig 4). Fig 4A plots the ratio obtained by iTRAQ (in log2) against the ratio obtained by SWATH for the same protein. We see that the ratios obtained by the two methods are well correlated (with a r^2^ of 0.726), which is quite more convincing than the correlation coefficient of 0.312 reported previously [17]. A linear regression model fitted to the data shows a near-null intersect (a value of 0.09) and a slope of 0.28, which confirms the compression effect often observed with iTRAQ [24]. This compression effect is also immediately visible when the ratios are presented in a volcano plot (Fig 4B) or in a density plot (Fig 4C).

The main drawbacks of the iTRAQ or TMT strategies are the underestimation of the fold change (compression effect) [24], especially for larger change [5]. Mixed MS/MS has been identified and evaluated as the major cause of iTRAQ /TMT underestimation [24]. In complex mixtures, two precursors can be selected in the same window for MS/MS. Since iTRAQ/TMT quantification relies on fragment reporter ions and those reporter ions have the same mass no matter which precursor is selected, the ratio will reflect the mean from the two precursors. Recently, MS3 performed on triple-stage mass spectrometers have been shown to almost completely eliminate interference [25].

In SWATH, many precursors are selected in the same window for MS/MS but the chances that all the fragment ions have the same mass is slim and the ratio is not affected as in iTRAQ.

Since quantification experiments often produce a classification into ‘over-expressed’ and ‘under-expressed’ lists, the direction of the fold-change is especially important. Indeed, a large difference in quantitation of log2 ratio of 0.5 and 3 (corresponding to ‘fold changes’ of 1.4 and 8 respectively) will have little impact on downstream gene-ontology and pathway analyses. But a comparatively smaller difference in quantitation between reported log2 ratios of -0.4 and 0.4 will have important implications for downstream analyses. Our results show that iTRAQ and SWATH agree on the ‘direction’ of the fold change over 92% of the time when differential ratios are chosen solely on the basis of a t-test p-value threshold. (See Fig 4D for a representation of the data that makes the changes in ‘fold change direction’ easy to notice). But the proportion of agreement on the change ‘direction’ between the two methods increases meaningfully if we use a value threshold in addition to the t-test to determine which ratios are ‘differential’. To choose this threshold, we fitted a normal distribution on the data and used 1 standard deviation as the value threshold for a ratio to be deemed differential. This meant that the absolute value of the log2 ratios had to be superior to 0.66 for SWATH and superior to 0.23 for iTRAQ. Using this 1 standard deviation value threshold, the agreement on the ‘direction of the change’ between iTRAQ and SWATH climbed over 98%.

It should be noted that SWATH seems to have a higher precision than iTRAQ since for the same p-value threshold SWATH reports differential ratios that are closer to 0 (see the area around 0 in Fig 4D). This has the surprising effect that SWATH reports more proteins to be differential than iTRAQ even though the SWATH experiment was based on a library composed of 30% less proteins than the number of proteins identified in the iTRAQ experiment. Our hypothesis about this phenomenon is that the increased statistical power of the SWATH method might come from an increased number of measure points used and a smaller variance between these measures. SWATH can use more measures than iTRAQ since for a given identified ms2 spectrum, the SWATH method allows many peaks (i.e. many measure points) to be used for the quantitation, whereas the iTRAQ method can use only one pair of peaks to compare two conditions. Furthermore, the SWATH quantitation will not be based on every possible measure points from a given spectrum, but on the X best ones (where X is determined by the user, and where ‘best’ is determined mainly by the intensity of the peaks). The fact that a possible measure point might not be used in the SWATH method based on a quality criteria whereas the iTRAQ method necessarily use its only measure might provide an advantage in statistical power to SWATH. With increasingly small ratios being statistically significant, it becomes evident that statistical significance can be very different from biological meaningfulness. This is why we recommend the use of two criteria: a criterion for the statistical significance (the p-value), and a criterion for biological meaningfulness (for example a threshold based on the distribution of the observed ratios).

To evaluate the compression effect of the SWATH quantitation, we spiked a bovine serum albumin digest into both conditions (IR and IS). 1fmol of BSA digest was spiked into 3 replicates of IS and 3 replicates of IR and 50 fmol of BSA was spiked in 3 other replicates of IS and IR. The SWATH experiment reported ratio values of 29.5 +/- 5 (mean +/- SD for the three replicates) (S1 Fig). This shows a small compression effect, since we expected a ratio of 50, but this compression effect is far less important than the one seen in iTRAQ [24]. Indeed, in a previous ABRF study (Association of Biomolecular Research Facility [26], myoglobin was spiked in a *E*. *coli* cell lysate at a real 10 fold ratio and the iTRAQ technique reported a ratio of 2.5.

### Gene ontology analysis

**Fig 5:**
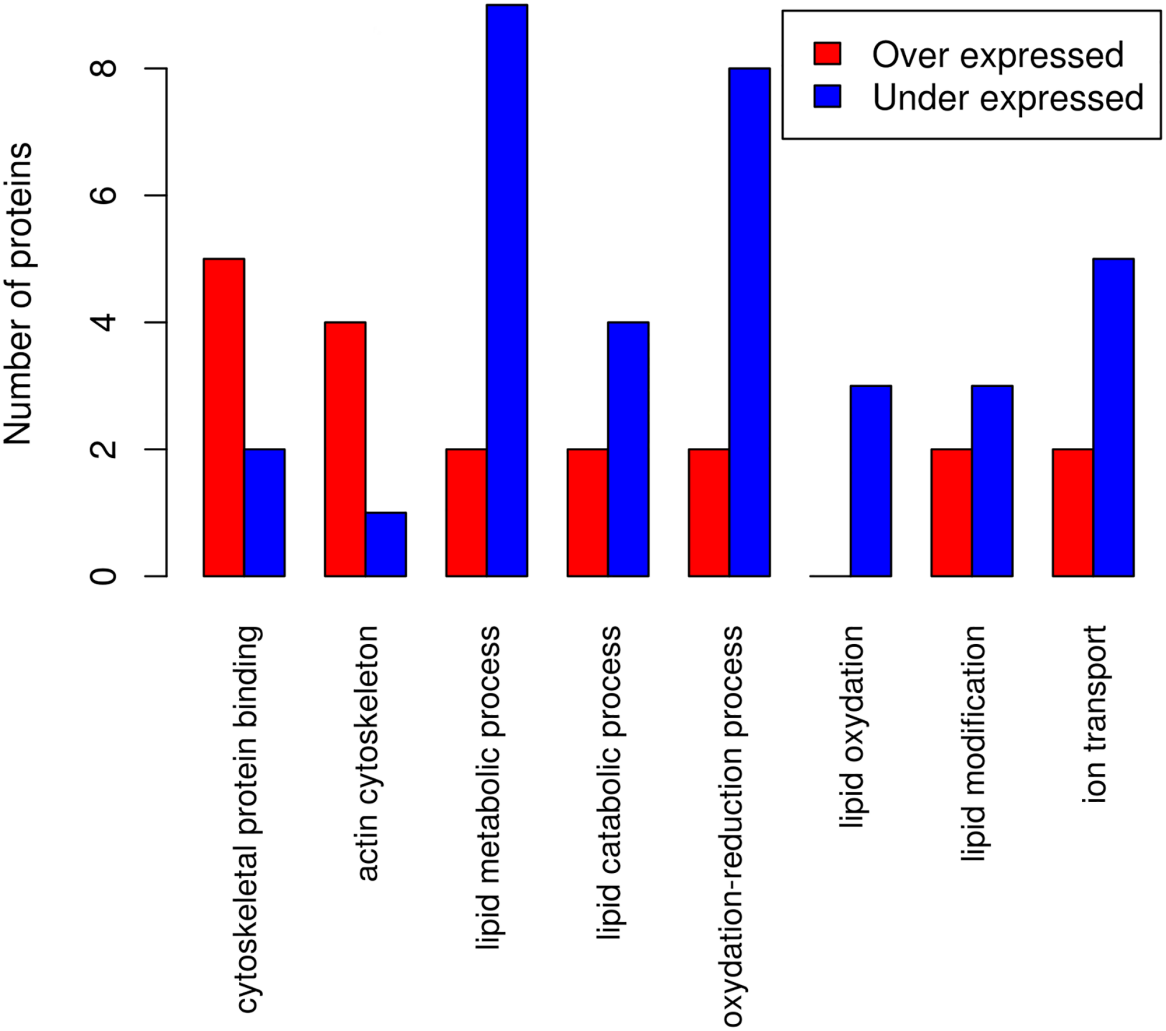
Functional analyses. Bioinformatic analyses reveal that proteins involved in lipid metabolic process and oxydation-reduction processed are mostly underexpressed in insulin resistant patients.

We used the proteins reported as differentially expressed by both the iTRAQ and SWATH experiments and performed Gene Ontology analysis using the three main ontologies (Cellular Component, Biological Process and Molecular Function). We found that proteins from the under-expressed list were far more likely to be associated with metabolic process and oxydo-reduction process than a random selection of proteins (p-values of 3e-5 and 7e-5 respectively) (Fig 5). It was previously demonstrated in a gene expression study that insulin resistance is associated with reduced expression of multiple genes encoding key enzymes in oxidative metabolism and mitochondrial function in skeletal muscle [27]. Our results in the duodenum seems to correlate with the one obtained in the skeletal muscle. We also found that many overexpressed proteins are related to the actin cytoskeleton (p-value for the overrepresentation of 1e-3). This is interesting in the present context given the documented role of the actin cytoskeleton in the regulation of insulin signals [28].

### Confirmation of protein expression

**Fig 6:**
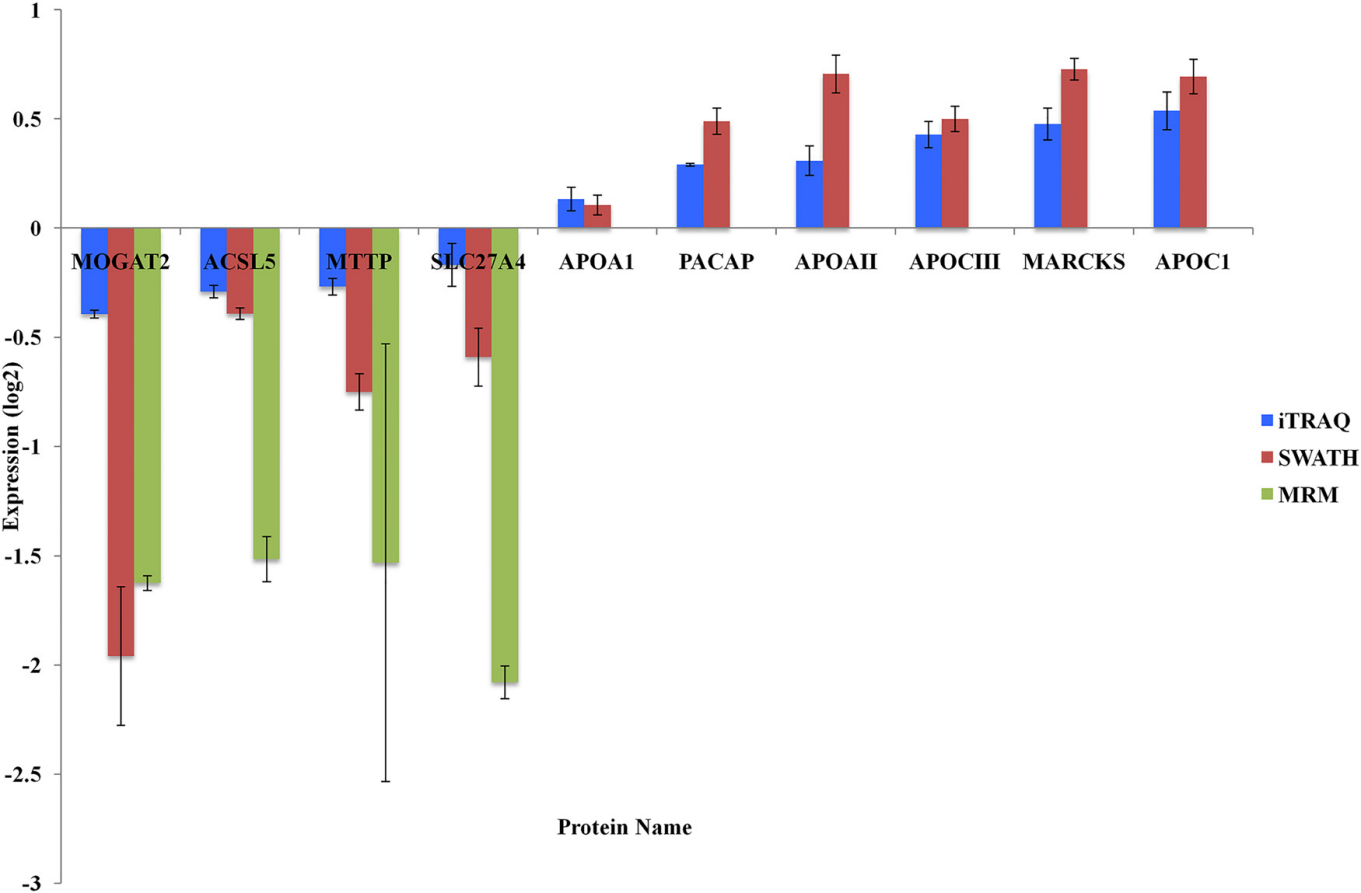
Differential expression of selected proteins. Results of the different quantitative proteomics analyses on selected proteins involved in lipid metabolism and insulin regulation.

One of the objectives of the study was to find regulated proteins associated with insulin resistance and lipid metabolism, Fig 6 shows the results for 10 chosen proteins. Four proteins were further assessed using MRM, which provides a more accurate measure of peptide abundance. The four proteins validated by MRM confirmed the iTRAQ and SWATH results. Unfortunately, due to lack of material, MRM results were not obtained for the other proteins.

However, one can observe that the ratio difference is greater in the MRM targeted experiment than in the SWATH and iTRAQ experiments. As discussed earlier, it is due to the compression effect observed with iTRAQ [24] and to some extent with SWATH (as the compression offect on spiked BSA in S1 Fig for example). The fact that only a few ions from the complex mixture are present during precursor selection in an MRM experiment (since they are targeted) in contrast with iTRAQ and SWATH where more interfering ions are present, contributes greatly to the better accuracy of the MRM results [24]. In addition, internal standards of known concentration were used in the MRM experiment to increase accuracy of the results [29].

### Biological significance of the results

Only a few human studies have examined the potential mechanisms underlying the oversecretion of intestinal lipoproteins in individuals with IR. Our previous study [4] has already shown by targeted proteomics that some key intestinal genes are down-regulated in dyslipidemic men with insulin resistance. However, only some targeted proteins were quantified at that time. Here, we used a proteomics discovery workflow to achieve a better understanding of the impact of insulin resistance on the duodenal protein expression.

Our results shows that proteins associated with lipid metabolism and catabolic processes are clearly underexpressed in insulin resistance, thus confirming the results obtained in our previous paper [4].

Indeed, both iTRAQ and SWATH results reveal that the protein MTTP (Microsomal Triglyceride transfer protein) and MGAT2 are down regulated in insulin resistant patients which has been shown previously by real-time PCR quantitation and MRM analyses [4]. Our results are in discrepancies with results obtained by other groups [30] working in diabetics insulin resistant patients and Phillips and colleagues [31] have also reported that diabetic subjects had significantly higher duodenal MTP mRNA levels than non-diabetic control subjects. The reason has been discussed previously [4] but in the fasted subjects with IR, the downregulation of intestinal MTP could be related to a greater inhibitory effect of insulin, whereas the oversecretion of intestinal lipoproteins observed in the constantly fed subjects with IR could reflect the fact that other factors, such as dietary lipid availability, are more important than the inhibitory effect of hyperinsulinism in determining MTP expression and lipoprotein secretion rates from the intestine.

ACSL5 expression was also decreased in IR (Fig 6). It has been previously shown [32] that ASCL5 is a target gene for SREBP-1c which is a transcription factor regulating the synthesis of sterols and that the mRNA expression of ACSL5 was decreased in diabetic animals. Our results confirms that ACSL5 seems to play a role in the response to insulin stimulus.

On the other hand, an increase in MARCKS levels (Fig 6), a protein which is involved in the regulation of insulin secretion is also observed in IR patients by both SWATH and iTRAQ. It has been previously shown [33] that insulin regulates MARCKS phosphorylation so there may be a link between insulin resistance and the level of unphosphorylated MARCKS.

The protein plasma cell-induced resident endoplasmic reticulum protein (also named PACAP) was found to be over-expressed in IR patients (Fig 6). It may have a role in the onset of insulin resistance. It has been found that MEDA-7 secreted protein (which is a protein isomer of PACAP) was able to decrease insulin-dependent glucose uptake in 3T3-L1 cells. The authors suggested that, Meda-7 upregulation could contribute to the progression of insulin resistance in adipose tissue [34].

## Conclusions

By using 2 different quantitative approaches, this study have shown that insulin resistance affects the proteins involved in lipid metabolism. A comparison of proteomics quantitation using iTRAQ and SWATH methods was presented. It was shown that results from both methods can have a high degree of correlation both in terms of actual quantitation and in terms of the ‘direction’ of the fold change. The methods also appear to have complementary aspects: for a given instrument time, the iTRAQ experiment will provide more protein identifications; however, the SWATH experiment can identify more differential ratios, being both more sensitive in the detection of small differential ratios and being able to cover a larger dynamic range. The differential proteins identified both confirm previous experiments (for example, the roles of MTTP, MGAT2 and ACSL5), and raises interesting new hypothesis, as with MARCKS and PACAP. Proteins of relevance to IR were mostly associated with metabolic and oxydo-reduction process and were found using both iTRAQ and SWATH-MS. These data also showed that SWATH-MS is a promising and compelling alternative to iTRAQ for protein quantitation of complex mixtures.

## Supporting Information

S1 Fig SWATH BSA response in the 12 samples. (TIFF)

S1 Table The 3886 proteins identified by iTRAQ with FDR < 1%. (XLS)

S2 Table The iTRAQ quantitative results from replicate one. (XLSX)

S3 Table The iTRAQ quantitative results from replicate two. (XLSX)

S4 Table The 2290 proteins identified in the SWATH library with FDR < 1%. (XLS)

S5 Table The SWATH quantitative results. (XLS)

S6 Table The quantitative results from the 208 common proteins between iTRAQ and SWATH with p-value < 0.05. (XLS)
