# Supplementary figures and images for: Evaluation of iTRAQ and SWATH-MS for the Quantification of Proteins Associated with Insulin Resistance in Human Duodenal Biopsy Samples
Source: PLoS One. 2015 May 7;10(5):e0125934. doi: 10.1371/journal.pone.0125934 (PMC4423961; doi:10.1371/journal.pone.0125934)

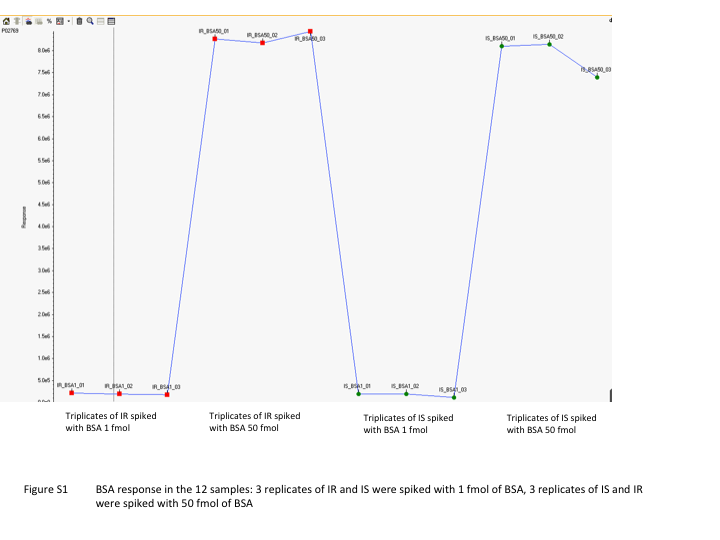

Supplement: S1 Fig — (TIFF) [file pone.0125934.s001.tiff]
